# Perceived dental treatment need among older Tanzanian adults – a cross-sectional study

**DOI:** 10.1186/1472-6831-7-9

**Published:** 2007-07-11

**Authors:** Anne N Åstrøm, Irene A Kida

**Affiliations:** 1 Centre for international health, UoB, Bergen, Norway; 2 Muhimbili University College of Health Sciences, Dar es Salaam, Tanzania; 3 Department of Oral Sciences-Community Dentistry, UoB, Bergen, Norway

## Abstract

**Background:**

Need perceptions for dental care play a key role as to whether people in general will seek dental care. The aim was to assess the prevalence of perceived need of problem based dental care, dental check-ups and any type of dental care. Guided by the conceptual model of Wilson and Cleary, the relationship of perceived need for dental care with socio-demographic characteristics, clinically defined dental problems and self-reported oral health outcomes was investigated. Partial prosthetic treatment need was estimated using a socio-dental approach.

**Method:**

A cross-sectional survey was conducted in Pwani region and in Dar es Salaam in 2004/2005. Information from interviews and clinical examination became available for 511 urban and 520 rural adults (mean age 62.9 yr).

**Results:**

51.7% (95% CI 46.2, 57.0) urban and 62.5 % (95% CI 53.1, 70.9) rural inhabitants confirmed need for dental check-up, 42.9% (95% CI 36.9, 48.9) urban and 52.7% (95% CI 44.5, 60.6) rural subjects confirmed need for problem oriented care and 38.4% (95% CI 32.4, 44.6) urban versus 49.6% (95% CI 41.8, 57.4) rural residents reported need for any type of dental care. Binary and ordinal multiple logistic regression analyses revealed that adults who reported bad oral health and broken teeth were more likely to perceive need for dental care across the three outcome measures than their counterparts. Socio-demographic factors and clinically defined problems had less impact. Based on a normative and an integrated socio-dental approach respectively 39.5% and 4.7% were in need for partial dentures.

**Conclusion:**

About half of the participants confirmed need for problem oriented care, dental check-ups and any type of dental care. Need perceptions were influenced by perceived oral health, clinically assessed oral problems and socio-demographic characteristics. Need estimates for partial denture was higher when based on clinical examination alone compared to an integrative socio-dental approach.

## Background

It has been suggested that perceptions of need for dental care play a key role as to whether people in general will seek dental care and that lack of need perceptions constitutes an important barrier for utilization of heath care services [1,2]. Reportedly, the main benefits of dental treatment relate to improved psychological and social well-being [3]. Thus, oral symptoms and functional and psychological impacts from oral conditions seems to be of great significance in the assessments of individuals' perceived need for dental care [3,4]. Need perceptions correlate, however, weakly with professionally determined need assessments based on clinical examination suggesting that people place importance on oral symptoms and functional impacts from oral diseases when evaluating their need for dental care services [review see [1]]. The extent to which oral symptoms and impairments play a role for need perceptions and whether or not dental care is sought, are modified by social and cultural factors, such as the ability to afford and attend dental care in the context of competing needs and other burdensome life circumstances [1]. Factors influencing perception of treatment needs and dental care utilization have been identified in several studies of older people from industrialized countries [1,4,5]. Public health care administrators are encouraged to design strategies for improved oral quality of life in the elderly [6]. Although the growth of the proportion of elderly is high in developing countries, few studies have investigated how socio-demographic-, clinical- and socio-behavioral characteristics contribute to the understanding of perceived need for dental care among older age groups of the sub-Saharan African population.

Adulyanon et al [7], Sheiham and Tsakos [8], Srisilapanan and Sheiham [9], and Srisilapanan et al [10] developed a method of estimating dental treatment needs by integrating oral health related quality of life (OHRQoL) measures with clinically based normative need estimates. This socio-dental system for need assessment represents a gradual integration process, including mainly three levels of assessment. Standard normative need assessment or level 1 is based solely on professionally judged clinical measures. Impact related need assessment, or level 2 relies on the integration of normative need with OHRQoL, considering such oral conditions that are unlikely to progress towards adverse health consequences in the absence of treatment. Propensity related need assessment or level 3 is calculated by integrating normative need assessment with impacts on OHRQoL and an individual's behavioral propensity. Following this socio-dental approach, Srisilapanan and Sheiham [9,10] reported that relying on normative methods (i.e. a clinical diagnosis) alone without integrating the psychosocial dimensions of oral health, seriously overestimated need for prosthetic treatment in older Thais. Accordingly, a normative measure of prosthetic treatment need estimated by converting clinical measures alone could probably be too high to be met in the Tanzanian context where the government's oral health care budget is inadequate to meet the increasing oral health needs of the population [11]. Previous studies considering tooth loss, psycho-social impacts and dissatisfaction with chewing ability have documented a substantial burden of oral diseases in older adults in Tanzania [12,13]. Chewing problems and dissatisfaction with chewing ability are prevalent and about 5 in 10 older adults have experience with at least one oral impact on daily performances. This prevalence of oral impacts is higher than those reported among comparable age groups in Norway, Great Britain and Greece but similar to that identified in Thailand [14-17].

### Purposes

**Figure 1:**
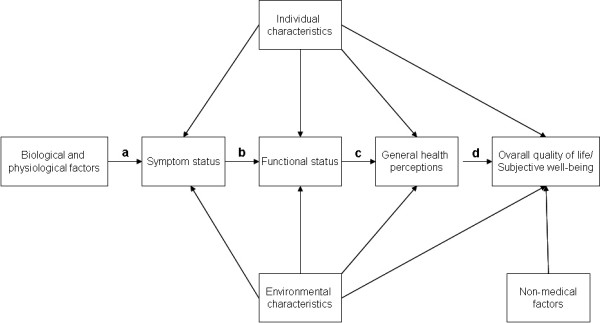
Conceptual model of patient outcomes adapted from Wilson and Cleary [18].

Focusing on Tanzanian adults 50 years and older, this study set out to assess the prevalence of perceived need for immediate problem oriented care, for dental check-ups and for any dental care (i.e. both problem oriented care and check-ups). Partial prosthetic treatment need was estimated using an integrated socio-dental approach [7]. In addition, the relationships of perceived need estimates with socio-demographics, clinically assessed dental problems and reported oral health outcome variables were investigated. Wilson's and Cleary's conceptual model (Fig 1) classifying oral health outcomes at five main levels; biological variables, symptom status, functioning, health perceptions and overall quality of life/well-being was applied to organize the independent variables and to guide the analyses [18]. As a test of the construct validity of this model and in accordance with its propositions, it was hypothesized that the responses to thirteen oral health outcome variables (observed indicator variables) could be explained by three correlated factors (latent variables) in terms of symptom status, functional disadvantage and general oral health perceptions.

## Methods

### Study area

A cross sectional survey was conducted in Pwani region, Eastern Tanzania and in the capital city of Dar es Salaam from November 2004 to June 2005. According to the 2002 population and housing survey in Tanzania, Pwani region has the highest number of older people 65 years and above in the country (7%). Dar es Salaam and Pwani region have a total population size of 2.5 million and 889,154, respectively. The corresponding figures for population densities are 1,793 and 27 persons per square km. The districts have drinking water with fluoride content of about 1 mgF/L.

### Sampling and procedure

Villages were selected from two rural districts (Kibaha and Bagamoyo) and one urban (Kinondoni) district in Pwani and Dar es Salaam, respectively. To obtain a sample of older adults of mixed socio-economic background, 107 pure urban (N = 59,688) villages and 96 pure rural villages (N = 26,520) were listed in Kinondoni and in Kibaha/Bagamoyo, respectively. A sample size of 1,200 older adults was calculated assuming a prevalence rate of tooth loss (≥1 missing tooth) of 50%, a precision of 4% and a design effect of 2 [19]. In the first stage, 10 pure urban villages (n = 6,290) and 10 pure rural villages (n = 3,729) were selected by systematic random sampling from the district village population lists. In the second stage, a total of 60 households were selected by systematic random sampling from each village selected in the first stage [for a more detailed description of the sampling procedure, see [12,13]]. The study was approved by the Research and Publication Committee at Muhimbili University College of Health Sciences, regional and district administration authorities, village leaders and by the ethical research committee in Norway (REK VEST). Informed consent was obtained from all participating subjects.

### Interview

A structured interview schedule was constructed in English and translated into Swahili before being administered in the field by two trained research assistants. Oral health professionals reviewed the interview schedule for semantic, experiential and conceptual equivalence. Sensitivity to culture and selection of appropriate words were considered. The interview schedule was piloted before administration. *Socio-demographics *were assessed in terms of place of residence, gender and age. *Frequency of dental attendance *during the previous 2 years – was coded (1) less than once and (2) once or more. *Having ever used tobacco products *was recorded (1) yes and (2) no. *Perceived need for immediate problem oriented care *was coded as (0) no and (1) yes. *Perceived need for immediate dental check-*up (examination and cleaning without any kind of treatment) was coded (0) no and (1) yes. A combined measure of *perceived need of any dental care *was constructed with the response categories (0) no perceived need, (1) perceived need for problem oriented care or dental check up, (2) perceived need for both problem oriented care and dental check up. Participants were asked to identify specific treatments needed by answering (1) yes or (0) no to the following questions: "pain release", "tooth extraction", "fillings", "partial dentures" and "full dentures". *General health status *was assessed by asking about general health problems such as obesity, cancers, heart disease, respiratory disease, trauma, diabetes, sight problems, blood pressure and arthritis. Answers were given as (1) yes and no (0). A sum index was constructed by adding the responses and further dichotomized revealing the categories (0) "no health problems" and (1) "at least one reported health problem".

The measures selected to operationalise symptoms, function and general health status within Wilson and Cleary's model are described below. *Symptom status *was assessed by asking subjects, whether they had experience with tooth pain and broken teeth during the previous two years. Response categories were given as (0) "no" and (1) "yes". Chewing difficulty was assessed by asking subjects how well they could chew a number of common Tanzanian food items. The response categories were (1) "well", (2) "less well", (3) "badly". The number of foods subjects could chew less well and badly was summed to give a chewing problem index score with a higher score indicating more difficulty with chewing [20]. *Functional disadvantage *was measured broadly using the eight item Oral Impact on Daily Performance, OIDP, inventory (e.g. During the previous 6 months – how often have problems with your teeth and mouth caused you any difficulty with eating, speaking, cleaning teeth, smiling, sleeping, emotional balance, work and social contact) [13]. *General oral health perceptions *were assessed by two items. Self-reported satisfaction with chewing ability was measured by one question using the categories (1) "very satisfied", (2) "satisfied", (3) "neither satisfied nor dissatisfied", (4) "dissatisfied", (5) "very dissatisfied" [dichotomized into (0) "satisfied" (original categories 1,2) and (1) "dissatisfied" (original categories 3,4,5)]. Self-reported oral health status was measured by one question and coded (1) "very good", (2) "good", (3) "average", (4) "bad", (5) "very bad" and further dichotomized into (0) "good" (original categories 1,2,3) and (1) "bad" (original categories 4,5).

### Clinical examination

A full mouth clinical examination, including 3^rd ^molars was conducted [21]. For a detailed description of the clinical examination procedure and scoring of clinical variables see [12,13,20]. *Tooth mobility *was assessed using a modified Miller's index [22]. An individual tooth mobility scores was defined as (1) "2 or more mobile teeth" (0) "less than 2 mobile teeth". *Caries experience *was assessed in accordance with the criteria described by the World Health Organization, WHO [21]. Caries experience was coded (0) "0–1 decayed teeth" and (1) "2–22 decayed teeth". *Number of anterior and posterior occluding units *was counted based on existing natural tooth contacts between maxilla and mandible in the bilateral posterior and anterior regions. A combined variable was constructed yielding the following categories; (1) "complete posterior and anterior occluding units (6AO & 10 PO), (2) "reduced anterior *or *posterior occluding units" (6 AO & 0–9 PO/0–5 AO & 10 PO) and (3) "reduced anterior occluding units and reduced posterior occluding units" (0–5 AO & 0–9 PO) [20].

### Reproducibility

Duplicate clinical examinations were carried out on a randomly selected sub-sample, considered to be representative of the study subjects. Duplicate assessments of the number of anterior occluding units were performed 2 weeks apart using clinical pictures of the study participants' anterior dentition. Analysis performed on the duplicate examination recordings gave weighted kappa statistics of 1.00 for missing teeth due to caries, decayed teeth and posterior occluding support. Kappa values of 0.77, 0.79 and 0.85 were obtained for mobile teeth, tooth loss due to other reasons than caries and the number of anterior occluding units, respectively.

### Statistical analyses

Cross-tabulation, chi-square statistics and Mc Nemar's test were used for bivariate analyses. Stepwise logistic regression using the logit-model with 95% CI (confidence interval) and ordinal regression were employed for multivariate analyses. The ordinal regression model is a preferable modeling tool that does not assume normality and constant variance but requires the assumption of parallel lines across all levels of the categorical outcome variable [23]. Confirmatory factor analysis, CFA, was employed to test the hypothesized 3-factor measurement model in line with Wilson and Cleary's framework. CFA specifies the relationships of observed indicator variables (e.g. OIDP, reported chewing problems) to underlying latent structures (e.g. symptoms, function and general oral health perceptions). The parameters of the model were estimated with maximum likelihood (ML) estimation using bootstrapping which has been advocated with non-normally distributed variables [24]. The adequacy of overall model fit was estimated using chi-square test statistics, root-mean-squared error of approximation (RMSEA), the Goodness of Fit Index (GFI), the Normed Fit Index (NFI) and the comparative fit index (CFI). In line with the conventional recommendations of Hu and Bentler [24], a good model fit was indicated by RMSEA less or equal to .06 and with a GFI, CFI and NFI greater or equal to .90. Data analyses were performed using SPSS (14.0), STATA (9.2) with survey commands to account for cluster design and AMOS (6.0) [25].

## Results

### Characteristics of participants

A total of 511 (participation rate 85.2%) urban and 520 (participation rate 86.7%) rural subjects between 50 and 100 yr (mean age 62.9, SD = 10.6, men 46.4%, no formal education 44.7%), completed an extensive personal interview followed by a full mouth clinical examination. The prevalence of tooth loss (≥1 tooth due to any reason calculated with the inclusion of edentulous people) was 85.5% (mean tooth loss 6.1, SD = 6.4) in urban areas and 82.1% (mean tooth loss 5.9, SD = 6.6) in rural areas. The prevalence of edentulous people was 0.6% (n = 6), which compares with what has been reported in similar age groups of Tanzanians previously [26]. A total of 20.7% had attended a dentist more than once during the previous 2 years, 55.6% had no experience with health problems and 77.1% said they did not use any tobacco products.

### Prevalence of reported need to see a dentist for various reasons

As shown in Table 1, 51.7% (95% CI 46.2, 57.0) urban and 62.5 % (95% CI 53.1,70.9) rural inhabitants confirmed need for dental check-up, whereas 42.9% (95% CI 36.9, 48.9) urban and 52.7% (95% CI 44.5, 60.6) rural subjects reported need for problem oriented care. A total of 38.4% (95% CI 32.4, 44.6) urban versus 49.6% (95% CI 41.8, 57.4) rural residents reported immediate need for *both *problem oriented care and dental check ups. The most frequently reported specific treatment need was pain relief. Perceived need for partial dentures was reported among 20.5 % (95% CI 17.3, 24.1) urban and 18.1% (95% CI 12.6, 25.1) rural residents.

**Table 1:** Percentages (n) who report immediate need to see a dentist for dental check-up, for immediate problem oriented dental care, for both dental check-up and immediate problem oriented dental care and for specific treatments by place of residence.

	Urban % (n)	Rural % (n)	Total
Need for dental check-up	51.7 (264)	62.5 (325)	57.1 (589)
Need for immediate dental care	42.9 (219)	52.7 (274)	47.8 (493)
Need for *both *check-up and immediate dental care	38.4 (196)	49.6 (258)	44.0 (454)
*Need for specific dental problems*			
Pain relief	30.3 (155)	42.5 (221)	36.5 (376)
Tooth extraction	17.4 (89)	25.5 (133)	21.5 (222)
Fillings	10.8 (55)	13.7 (71)	12.2 (126)
Partial dentures	20.5 (105)	18.1 (94)	19.3 (199)
Full dentures	2.7 (14)	3.7 (19)	3.2 (33)

### Model of oral health outcomes- construct validity

The 3-factor model allowing for cross loading of 3 OIDP indicator variables on general oral health perceptions indicated moderately acceptable fit with the GFI, IFI, NFI and CFI indices (>.90). The RMSEA was above the acceptable level (.08) and the chi-square was statistically significant (p < 0.001). All bootstrap item loading with 90% CI (bias corrected percentile method) were reasonable, statistically significant. The observed variables explained 71% (90% CI 62–80), 62% (90% CI 53–72) and 43% (34–55) of the variance in functional disadvantage, oral health perceptions and symptom status, respectively. Alternative 2-factor and 1-factor models were tested yielding highly statistically significant chi-square statistics and high RMSEA values. In addition none of the other fitting criteria were met, indicating that a 3-factor model fitted the data better than either a 2-factor or a 1-factor model.

### Correlates of perceived need

The percentage distributions of participants' socio-demographic-, clinical-, symptom,-function-, and oral health perception variables are shown in Table 2. The adjusted ORs from stepwise binary logistic regression analyses of subjects who confirmed need for problem oriented care and dental check-ups according to socio-demographic-, clinical and oral health outcome variables are depicted in Table 2 (Model I) and Table 3 (Model II). Age, gender and place of residence were entered into step I providing a Nagelkerke's R^2 ^of 0.07 (P = 0.001) and 0.06 (P = 0.000) in Model I and Model II, respectively. Entering clinical variables in step II, raised Nagelkerke's R^2 ^to 0.11 in Model I and 0.08 in Model II. Variables reflecting symptom status entered in step III raised the Nagelkerkes R^2 ^to 0.26 in Model I and to 0.16 in Model II. OIDP and general oral health perceptions entered in step IV and V raised the Nagelkereks R^2 ^to 0.32 and 0.40 in Model I and to 0.23 and 0.28 in Model II (P = 0.001). In the final Model I (Table 2), subjects who reported need for immediate problem oriented care were less likely to be 70 years and older (OR = 0.6) and to be females (OR = 0.6). They were more likely to have attended a dentist (OR = 1.5), to have reduced occluding support (OR = 1.8), broken teeth (OR = 1.9), at least one oral impact (OR = 2.1), being dissatisfied with chewing ability (OR = 1.8) and dissatisfied with oral health status (OR = 4.6). In the final Model II (Table 3), females were less likely to report need for check ups (OR = 0.5). Subjects who reported need for dental check-ups were more likely to have experienced broken teeth (OR = 1.9), had at least one OIDP (OR = 2.5), were dissatisfied with chewing ability (OR = 1.9) and were dissatisfied with their oral health status (OR = 2.7).

**Table 2:** Model I: Perceived need for immediate problem oriented care regressed on clinical and non-clinical factors Percent confirming need (n), adjusted OR and 95% Confidence intervals, CI.

Determinant – overall % in population (100%)	% (n)	P value	Adjusted OR	95% CI
**Step I (Sociodemographics)**				

Age: 50–59 (n = 454/44.0%)	52.0 (236)		1	
60–69 (n = 303/29.4%)	43.9 (133)		0.7	0.4–1.0
70+ (n = 274/26.6%)	45.3 (124)	0.056	**0.6**	**0.3–0.9**
Male (n = 478/46.4%)	50.6 8242)		1	
Female (n = 553/53.6%)	45.4 (251)	0.104	**0.6**	**0.4–0.9**
Urban (n = 511 49.6%)	42.9 (219)		1	
Rural (n = 520/50.4%)	52.7 (274)	0.002	1.2	
Dental attendance: never (n = 797/77.3%)	42.9 (342)		1	0.8–1.7
>once (n = 234/22.7%)	64.5 (151)	0.000	**1.5**	**1.1–2.2**
**Step II (Clinically assessed dental status)**				
Full PO & AO (n = 125/12.9%)	32.8 (41)		1	
Reduced AO or PO (n = 460/47.6%)	46.5 8214)		**1.7**	**1.1–2.0**
Reduced PO & AO (n = 382/39.5%)	55.2 (211)	0.000	**1.8**	**1.1–2.9**
Decayed: 0–1 teeth (n = 508/49.3 %)	41.5 (211)		1	
Decayed: 2–22 teeth (n = 523/50.7%)	53.0 (282)	0.000	0.9	0.6–1.2
Tooth mobility: <2 teeth (n = 830/80.5%)	45.5 (378)		1	
Tooth mobility: ≥2 teeth (n = 201/19.5%)	57.2 (115)	0.004	0.7	0.6–1.4
**Step III (Symptom status)**				
Experience pain: no (n = 456/44.2 %)	27.4 (125)		1	
yes (n = 575/55.8%)	64.0 (368)	0.000	1.3	0.9–1.9
Experience broken teeth: no (n = 636/61.7%)	38.4 (244)		**1**	
Yes (n = 395/38.3%)	63.0 (249)	0.000	**1.9**	**1.3–2.6**
Chewing problem: no (n = 617/59.9%)	37.6 (232)		1	
Chewing problem: ≥1 food (n = 413/40.1%)	63.0 (260)	0.000	1.2	0.8–1.7
**Step IV (Functional disadvantage)**				
OIDP: none (n = 442/43.2%)	24.4 (108)		**1**	
≥1 (n = 582/56.8%)	65.6 (382)	0.000	**2.1**	**1.5–2.9**
**Step V (General oral health perceptions)**				
Satisfied chew ability: yes (n = 766/74.3%)	37.7 (289)		**1**	
no (n = 265/25.7%)	77.0 (204)	0.000	**1.8**	**1.2–2.8**
Oral status good: yes (n = 663/64.3%)	30.2 (200)		**1**	
no (n = 368/35.7%)	79.6 (293)	0.000	**4.6**	**3.1–6.7**

**Table 3:** Model II: Perceived need for dental check-ups regressed on clinical and non-clinical factors. Percentages (n) confirming need, adjusted Odds ratios, OR, and 95% Confidence interval, CI

Determinant-overall % in the population (100%)	% (n)	P value	Adjusted OR	95% CI
**Step I (sociodemographics)**				

Age: 50–59	58.6 (266)		1	
60–69	57.1 (173)		0.8	0.6–1.3
70+	54.7 (150)	0.597	0.2	0.1–1.1
Male	63.0 (301)		1	
Female	52.1 (288)	0.000	**0.5**	**0.4–0.7**
Urban	51.7 (264)		1	
Rural	62.5 (325)	0.000	1.5	**1.1–2.1**
Dental attendance: never	42.9 (342)		1	
>once	64.5 (151)	0.000	1.3	0.9–2.0
**Step II (Clinically assessed oral status)**				
Full PO & AO	49.6 (62)		1	
Reduced AO or PO	58.5 (269)		1.5	0.9–2.4
Reduced PO and AO	59.9 (229)	0.119	1.1	0.6–1.9
Decayed: 0–1 teeth	54.5 (277)		1	
2–22 teeth	59.7 (312)	0.102	0.7	0.5–1.1
Tooth mobility: <2 teeth	45.5 (378)		1	
≥2 teeth	57.2 (115)	0.004	0.9	0.6–1.4
**Step III (Symptom status)**				
Experience pain: no	43.0 (196)		1	
yes	68.3 (393)	0.000	1.1	0.7–1.4
Exp broken teeth: no	49.5 (315)		**1**	
yes	69.4 (274)	0.000	**1.9**	**1.3–2.6**
Chewing problem: no	52.2 (322)		1	
≥1 food	64.4 (266)	0.000	0.7	0.5–1.0
**Step IV (Functional disadvantage)**				
OIDP: none	36.9 (163)		**1**	
≥1	72.3 (421)	0.000	**2.5**	**1.8–3.5**
**Step V (General oral health perceptions)**				
Satisfied chew ability: yes	49.3 (378)		**1**	
: no	79.6 (211)	0.000	**1.9**	**1.3–2.9**
Oral status good: yes	44.3 (294)		**1**	
no	80.2 (295)	0.000	**2.7**	**1.8–4.0**

Ordinal logistic regression analyses (without variance estimation since no test for the parallel regression assumption was available with STATA survey command) were performed with perceived need for any dental care as categorical dependent variable. The results showed a similar pattern of relationship with the same set of independent variables as employed in the models presented in Table 2 and 3. Subjects, 70 years and older compared to 50–59-year olds and females compared to males were less likely to perceive need for any dental care. The corresponding ORs were 0.7, 95% CI 0.4–0.9 and OR = 0.5, 95% CI 0.4–0.7. Subjects reporting broken teeth (OR = 1.9, 95% CI 1.4–2.7), oral impacts on daily performance (OR = 2.3, 95% CI 1.7–3.2), dissatisfaction with chewing ability (OR = 1.9, 95% CI 1.3–2.7) and bad oral health status (OR = 3.9, 95% CI 2.7–5.6) were more likely to perceive need for any dental care.

### Normative-, impact-, and behavioral propensity related need for partial dentures

A total of 39.5% (382/967) fulfilled the criteria for clinically assessed normative treatment need for partial dentures in terms of having reduced occluding support (0–5 AO & 0–9 PO). A total of 22% (215/967) fulfilled the criteria of a modified normative treatment need in terms of being without a history of serious health problems and at the same time having reduced occluding support. In turn, 13.8% (132/960) fulfilled the criteria for impact related treatment need, i.e. having a modified normative need and reporting oral impacts. Finally, 4.7 % (45/960), 2.8% in urban and 6.9% in rural, fulfilled the criteria for having propensity related need for partial dentures, i.e. having impact related need and at the same time confirming no use of tobacco products. Mc Nemar test revealed statistically significant difference between the two normative need estimates (39.5% and 22%) and that taking into account socio-behavioral factors (4.7%) (P < 0.001).

## Discussion

According to theory [27] and empirical evidence [28], perceived need for dental care is strongly associated with utilization of dental health care services. Thus, an understanding of people's need perceptions is important for effective planning and implementation of such services. Overall, the present results based on confirmatory factor analysis lend support to the construct validity of Wilson and Cleary's conceptual framework as applied to older Tanzanian adults, suggesting that responses to oral health outcomes might be explained by three conceptual domains; symptom status, functional disadvantage and general oral health perceptions. According to the indices of GFI, NFI, CFI and RMSEA, the 3-factor measurement model fitted the current data moderately well, indicating inappropriate operationalisation of domain variables and need of further modifications of the measurement model. Using each single indicator variable of oral health outcomes in the multivariate analyses together with clinically assessed problems and socio-demographics, clarified their relative importance as correlates of perceived problem oriented need, perceived need for dental check-ups and perceived need for any type of care. Consistent with previous findings from industrialized countries [1,4,5], decreased number of occluding support, worse oral symptom status, deteriorated oral function and dissatisfaction with oral health status/chewing ability were all positively associated with perceived need for dental care. This suggests that a full understanding of need perceptions in older Tanzanians cannot be captured by clinical assessments alone.

The present study was confined to information obtained from 1031 dentate and edentate adults, 50 years and older living in urban Kinondoni and rural Kibaha/Bagamoyo districts of Tanzania. A comparison of the sex and age characteristics of the study participants and census data from the target population indicates that the former are broadly representative of the latter, except for a slight over sampling of females in urban areas [26]. Of the subjects investigated, 47.8 %, 57.1 % and 44.0 % confirmed need for problem oriented care, dental check-ups and for any type of dental care, respectively. The prevalence of various categories of self-reported treatment need ranged from full dentures (3.2%) to pain relief (36.5%), reflecting those conditions that have been recorded as main reasons for dental attendance in Tanzania [29-31]. Evidently, most of the oral health care services in Tanzania are associated with relief of pain and discomfort [31]. The present figures of perceived treatment needs compare to those of older age groups in Sri Lanka where need for dental care and dentures were estimated to 43% and 53% [32], but are clearly below the proportion of 64.8% reported in older Canadians [33].

Despite the moderate level of oral disease, the prevalence of perceived need for problem oriented care and dental check-ups overestimated that of loss of occluding support and tooth mobility, but underestimated that of tooth decay. The observed discrepancy with reduced occluding support and tooth mobility confirms previous evidence that symptoms, impaired function and general oral health perceptions emanating from those oral conditions are substantial among older Tanzanians [13]. Consistent with previous results, these findings suggest that normatively assessed treatment need differs from older adults' assessment of their oral health status and from their perceived need for dental care [1,2,4,34]. Compared to a more rational integrated need estimate (4.7%) based on a socio-dental approach, the clinically based normative method seriously overestimated treatment needs for partial dentures (39%) in older Tanzanians [8-10]. Similar findings were reported among older Thais (10). Notably, the integrated need estimate did not match self-reported need for partial dentures and should be interpreted with caution as condition specific oral impacts (oral impacts emanating from loss of occluding support) were not available and since financial problems could not be integrated into the estimate of treatment need.

No statistically significant interaction effects were observed between place of residence (urban/rural) and the independent variables upon perceived need for dental care. Thus it was decided to use the pooled data for urban and rural residents in the logistic regression analyses.

According to the results from logistic regression analyses, 9 of the 13 explanatory variables included were significantly associated with perceived need for problem oriented care. The comparable number of variables for the two other measures of perceived need was 6. Perceived need for dental check-ups was defined as need for examination and cleaning without any treatment and thus the difference in explained variance in Model I and Model II might highlight the importance of discriminating between various types of need perceptions. Reduced number of occluding units increased the perceived need of problem oriented care, whereas self-reported broken teeth contributed both to perceived problem oriented care and to perceived need for dental check-ups. Conditions like broken teeth tend to be apparent to individuals and subjects with broken fillings are likely to be regular dental attendees [35,36]. Whereas clinically assessed oral problems and pain experience were positively associated with measures of need perceptions in the bivariate analyses, they usually did not remain statistically significant in the multivariate analyses. This suggests that their effects were mediated by variables within the domains of functional disadvantage and general oral health perceptions as predicted by the model of Wilson and Cleary [18].

Previous use of dental care services was positively associated with perceived need for problem oriented care and dental check ups in the bivariate analyses, but did only approach statistical significance when accounting for the other independents in the models. Perceived need has been associated with dental care utilization in a number of cross-sectional and longitudinal studies [2,37]. However, the prevalence of dental care utilization was less satisfactory among elderly in Tanzania with about 23% confirming utilization in the past 2 years. Moreover, 43% did not make a dental visit in spite of perceiving a need for problem oriented dental care. This finding should be interpreted in light of the limited access to oral health care services and the lack of high quality services in Tanzania where only 95 qualified dentists (dentist to population ratio 1:300.000) and 200 rural medical aides are available for the total Tanzanian population [38-40]. Dental diseases have low priority compared to other life-threatening conditions and the number of patients utilizing dental care services have declined since cost sharing was introduced through user fees for the health services provided by the Government [31]. Cost of dental treatment has been identified as a major barrier to obtaining health care by older individuals in Tanzania and most so for individuals at the lower end of the income distribution [39]. Although the number of missing teeth increases with increasing age in older Tanzanians [12], subjects 70 years and older were less likely to perceive need for problem oriented care compared to their younger counterparts. This finding agrees with those reported by other investigators [4,32,41]. The inverse relationship with increasing age that emerged when allowing for the effects of other variables might reflect age related changes in expectations, attitudes and values towards oral health care.

## Conclusion

Despite a moderate level of oral diseases, the prevalence of perceived need for problem oriented care, dental check-ups and any type of care was substantial among older Tanzanians. Moreover, socio-demographics and reported oral health outcome variables alongside clinical indicators were important correlates of need perceptions across the three outcome measures employed in this study. Results from confirmatory factor analysis suggest that the observed oral health outcome variables could be described in terms of three independent domains as proposed by Wilson and Cleary. Some fit indices were however below the optimal level suggesting a need for further development of the measurement model utilized. A normative need estimate based on the number of occluding units seriously overestimated treatment need for partial dentures when compared to a socio-dental approach. Knowledge of the factors influencing need perceptions is important for effective planning and implementation of oral health care services in Tanzania. However, improvements in utilization and delivery of dental health care services in this population will depend on the general social and economic development as well as on community based health promotion activities.

## Competing interests

The author(s) declare that they have no competing interests.

## Authors' contributions

ANÅ: conceived of the study, designed the study, performed statistical analyses and had the main responsibility for writing the paper

IAK: Principle investigator was actively involved in the planning and conduct of the study. Has been actively involved in manuscript writing

Both authors have approved the final manuscript

## Pre-publication history

The pre-publication history for this paper can be accessed here:


